# The Longitudinal Effects of Parent–Adolescent Digital Communication on Depression and Anxiety Symptoms

**DOI:** 10.1016/j.jadohealth.2025.05.025

**Published:** 2025-08-21

**Authors:** Bradley M. Trager, Ina M. Koning, Layla M. Rainosek, Suzanne M. Geurts, Regina J. J. M. van den Eijnden, Helen G. M. Vossen

**Affiliations:** a Department of Psychological Science, Loyola Marymount University, Los Angeles, California; b Department of Educational and Family Studies, Clinical Child and Family Studies, VU Universiteit Amsterdam, Amsterdam, Netherlands; c Department of Interdisciplinary Social Science, Youth Studies, Utrecht University, Utrecht, Netherlands; d Department of Education and Pedagogy, Clinical Child and Family Studies, Utrecht University, Utrecht, Netherlands

**Keywords:** Adolescence, Parenting, Parent–adolescent digital communication, Depression symptoms, Anxiety symptoms

## Abstract

**Purpose:**

Digital communication between parents and adolescents is prevalent. This study explored the overall frequency of digital communication and frequency of exchanging specific content between parents and adolescents as predictors of adolescents’ depression and anxiety symptoms.

**Methods:**

Participants were 188 mother–adolescent and 137 father–adolescent dyads from the Netherlands. Parents (mothers/fathers: *M*_age_ = 46.17 of 48.55) and adolescents (*M*_age_ = 13.88) each completed 2 surveys 1 year apart.

**Results:**

Results from the study suggest that the nature of the digital communication content rather than its overall frequency plays a crucial role in adolescents’ mental health outcomes. Sharing personal experiences with mothers was found to be protective against depression symptoms for both sons and daughters, while practical exchanges with mothers were protective for sons but not daughters. Conversely, frequent practical exchanges with fathers were associated with higher levels of anxiety symptoms. Furthermore, sharing humorous content was associated with higher levels of depression symptoms.

**Discussion:**

Findings suggest that the type of digital communication, rather than its overall frequency, plays a crucial role in adolescent mental health. Future studies are needed to better understand how these types of communication work in conjunction with in-person communication.

Adolescence is a critical developmental period marked by significant physiological, psychological, and social changes. Understanding the factors influencing the mental well-being of adolescents has never been more necessary, with studies over the past 2 decades indicating that the prevalence of psychological disorders among adolescents continues to rise unabated [1–5]. Depression and anxiety are arguably the two of the most common psychological disorders among adolescents, with the global prevalence estimated to be at 6.5% and 13.4%, respectively [6]. The COVID-19 pandemic is also likely to have contributed to an increase in mental health problems among this population [7–9]. Given the long-term outcomes of adolescent depression and anxiety, including poorer physical health, substance use issues, and higher likelihood of future unemployment, among others [10–13], identifying factors that help mitigate adolescent depressive and anxiety symptoms early on may have meaningful implications for those affected.

Numerous factors, including genetic predispositions, personal microbiomes, and environmental influences, play a role in the onset and exacerbation of these mental health challenges, which frequently emerge during adolescence [14–18]. Among the various environmental factors that influence adolescents, parents play a primary role, with their behaviors contributing to the development of depressive and anxiety symptoms that can persist into adulthood. According to the Family Systems Theory [19], family functioning is fundamentally shaped by communication, as all behaviors are rooted in the intent of the actor and interpreted by observers, making them inherently communicative. This process can either reinforce or mitigate problematic and adaptive behaviors over time [19]. In accordance with the Family Systems Theory, parent–child communication can influence childhood internalizing behaviors in both positive and negative ways. Certain behaviors (e.g., less warmth, less autonomy support, aversiveness, and overinvolvement) may act as risk factors, while others (e.g., authoritative parenting, parental monitoring, and healthy communication) have been firmly established as protective against internalizing mental health issues [20–23]. Given the strong influence of parent–child communication on adolescent mental health, studying family communication is essential for developing holistic interventions aimed at preventing and treating depressive and anxiety symptoms.

## Digital communication

In today’s digital age, technological advances, particularly smartphones, have significantly reshaped parent–adolescent communication, with potential implications for mental health and well-being. While adolescents frequently communicate digitally with their peers, many also engage in regular digital communication with their parents [24,25]. Digital communication can encompass a wide range of activities made accessible through the use of smartphones, including texting, calling, voice notes, and social media interactions. There appears to be a moderately high level of digital contact between parents and adolescents. For instance, ~95% of Dutch adolescents have reported digital communication (i.e., Snapchat, WhatsApp, and Instagram) with their parents, with ~15% reporting digital contact on a near daily basis [26]. Even as children transition into young adulthood, this pattern persists, with over 70% of parents communicating with their slightly older young adult children digitally every week and a substantial portion exchanging texts or calls daily [27]. While in-person interactions likely remain the primary mode of communication while children live at home, digital communication appears to be a ubiquitous supplement.

There is a growing literature that suggests that communicating digitally with a parent can enhance the relationship between parents and adolescents by facilitating opportunities for social support and day-to-day communication, even when physically apart [28,29]. It also serves as a tool for parents to monitor their adolescents, with over 90% utilizing texting for this purpose [30]. While digital communication may result in some negative parenting behaviors (e.g., overmonitoring, helicopter parenting, or other autonomy-inhibiting behaviors [31–34]), these tendencies are likely a reflection of an individual parent’s parenting style. That is, parents who engage in unhealthy behaviors during face-to-face communication may be more likely to continue such behaviors in a digital setting. Fortunately, the aforementioned negative parenting behaviors are not the norm, and research has shown that digital communication in and of itself with parents is not usually considered autonomy prohibiting [26,31]. Moreover, research indicates that on days when adolescents frequently engage with their parents through digital means (e.g., seeking support, checking in) over a 14-day period, they also report more positive offline interactions with them [35]. Additionally, the same study found that daily digital communication was not associated with same-day depression (i.e., feeling sad, tired, or lonely) or anxiety (i.e., feeling worried) [35]. One potential reason why digital communication was not shown to have effects on same-day depression or anxiety symptoms is that its impact of the mental health outcomes may be due to the cumulative effects of the communication [36], as opposed to more brief, fleeting influences that may not be consistent across wider time spans. In addition, the development of depressive and anxiety symptoms may take years [37]. Thus, assessing frequency of overall digital communication over a longer period than previously examined may yield different effects on depression and anxiety symptoms.

## Content of Digital Communication

Another potential factor that could play a role in shaping adolescents’ mental health outcomes communication is the content of the parent–adolescent digital communications. There are 4 main content areas that are under-researched within the realm of digital communication that may yield a more comprehensive approach towards future parent interventions for children struggling with depressive and anxiety symptoms.

### Exchanging practical information.

The first such area being the utility of exchanging practical information via digital methods. Microcoordination, or communication dealing with day-to-day care and social coordination, is the most common form of digital communication [26] and may suggest a level of daily parental involvement and care in their child’s life. Questions relating to their child’s current location or what time they will be home falls within the scope of parental monitoring, which can be highly beneficial for adolescents on a wide range of outcomes, including both internalizing (e.g., mental health problems and somatic issues) and externalizing issues (e.g., substance use, risky sexual behavior, and lower school achievements). [38–40] As such, the exchange of practical information via digital methods may prove beneficial for children’s wellbeing.

### Sharing experiences.

A second content area deals with sharing experiences via digital communication. Self-disclosure of feelings and personal experiences by parents can help facilitate an environment that welcomes open and honest discussions and builds trust within familial relationships; when adolescents feel supported by their parents and engage in frequent, open communication with each other, anxious and depressive symptoms can be reduced [41,42]. Additionally, children sharing their experiences with their parents may also relate to seeking support, and those with the highest mental health needs are also the ones who tend to seek out digital support the most [35]. Online self-disclosure has also appeared to benefit highly anxious adolescents more than offline interactions [43]. Disclosing feelings through remote conversations may then invite opportunities for connection or initiate a request for support that otherwise would have been inaccessible and can therefore be protective against negative mental health outcomes.

### Use of humor.

The third content area that needs further research is the utilization of humor via digital communication. Research on the use of humor as a coping strategy within parental communication has been mixed, with some studies linking the use of humor between parent and child with fewer depressive/anxiety symptoms in adolescents in general [44], and others finding a similar trend for girls but not boys [22,45]. Overall, the use of humor in parent–child communication may be indicative of protective effects against depression and anxiety, and it is worth further study to continue to parse out the intricacies of this relationship within the realm of digital communication.

### Expression of feelings.

Finally, the fourth content area, the expression of feelings, is most supported within the body of research relating to parent–child communication. Extant research has been done supporting the notion that emotional expression is beneficial within families [46–48]. Parents who establish strong relationships with their children through emotional support are more responsive to their child’s needs, and subsequently this support is related to greater well-being and psychological adjustment later in life [48]. Digital discussions around feelings would likely offer the same benefits to adolescents, while also presenting the opportunity to start conversations remotely instead of limiting these interactions to in-person settings only.

## Mother– and father–adolescent communication

As described above, research on parent–adolescent digital communication suggests that these exchanges can foster a supportive and responsive environment that may help buffer against mental health challenges. However, less is known about the effects of mother– and father–adolescent digital communication on depression and anxiety symptoms over time. While the gender gap in parenting roles has narrowed, mothers still tend to be more involved in their children’s lives, providing greater emotional support, day-to-day involvement, parental control, and warmth [47,49,50]. A recent meta-analysis also found that mothers are more likely to adopt authoritative parenting styles, whereas fathers are more likely to exhibit authoritarian tendencies [50]. Despite these differences, many studies combine mother and father scores due to challenges in adequately powering analyses to detect parental differences. This approach, while practical, may obscure meaningful distinctions in parenting behaviors that could inform more targeted interventions. However, studies that have been able to examine mother- and father-specific communication suggest that their effects on adolescent depression and anxiety may differ [36]. These findings underscore the importance of research, such as this study, in further clarifying how maternal and paternal digital communication might be associated with adolescent mental health and mood states.

## Current study

This study addresses a critical gap in the literature by exploring the longitudinal associations between the frequency of patterns of parent–adolescent digital communication on adolescent mental health (i.e., depression and anxiety symptoms) over a 12-month period. The effects of the content of these communications, including the exchange of practical information, personal experiences, humor, and emotional expressions, were also examined to determine if what parents and their adolescents are communicating matters more/less than the general act of communicating with each other. We also examined digital communication with mothers and fathers separately to identify whether both mothers and fathers might influence their adolescents’ mental health via digital communication. This is important given the understanding that mothers and fathers may exhibit different parenting behaviors [32], which may, in turn, differentially affect their adolescent’s mental health.

Additionally, we also examined adolescent birth sex as a moderator of frequency of overall digital communication and specific communication content given that previous research suggests that there are differences in communication patterns that emerge during adolescence between parents and sons and parents and daughters (e.g., boys are less open when communicating with parents than girls [51]). Such differences may contribute to sex differences in the prevalence and manifestation of depression and anxiety symptoms found between boys and girls (i.e., these internalized conditions are typically higher in girls than boys [14]). Finally, to partially account for the familial transmission of depression and anxiety, we included self-reported parental depression and anxiety symptoms to help isolate the potential effects of digital communication patterns on adolescent outcomes.

Based on the research described thus far, we hypothesize that digital communication, irrespective of the content, will have a protective effect against depression and anxiety symptoms in adolescents. All other effects examined in this study are exploratory, intended to uncover nuanced relationships within parent–adolescent digital interactions.

## Methods

### Procedure and participants

The data used in the current study are part of a larger longitudinal study on parenting and digital media use among youth (Digital Family project [52]). Data from the second and fourth wave (now referred to as T1 and T2 respectively) were used since online parent–adolescent communication was only included from wave 2 onwards. T1 data were collected from May through July 2021 and again 12 months later (T2) among Dutch families that were recruited using various means including social media channels, newsletters of schools and sport clubs, paper flyers, and word of mouth. Families could participate in this research project with at least one parent or main caregiver (hereafter referred to as parent) and one child, with a maximum of 2 parents and 2 children. Adolescents and their parents were each required to have access to a smartphone to participate in this study and were asked to complete online questionnaires individually at home. At the beginning of the questionnaire, participants provided active informed consent. Active parental informed consent was obtained through the registration form. Completing the questionnaire took 30–45 minutes. Each participant received a €5 gift card, and families had the opportunity to win a €100 gift card. The Digital Family project was approved by the Ethics Committee of the Faculty of Social and Behavioral Science at Utrecht University (FETC20–192).

Only participants who provided complete T1 and T2 data were included in the analyses. Twenty-four mothers and 29 fathers were excluded at the outset—not because they did not participate at T1, but because they responded “never” on the T1 digital communication frequency item. This response precluded them from receiving the follow-up communication items, resulting in missing data at T1 for those variables (see the Measures section). After these exclusions, there were 334 mother–adolescent and 248 father–adolescent dyads at T1 (226 adolescents had data for both parents). Of these, 188 mother–adolescent and 137 father–adolescent dyads also completed T2. In total, 195 adolescents had complete T1 and T2 data from at least one parent and were included in the analytic sample (130 adolescents had data from both parents, 58 from mothers only, and 7 from fathers only).

At T1, adolescents were on average 13.88 years of age (standard deviation [*SD*] = 2.05; min = 10, max = 18), and 57.9% were girls (*n* = 113) and almost all adolescents were born in the Netherlands (98.3%). Regarding educational level of the adolescents, 21.8% were in primary school, 11.1% in prevocational education (i.e., all so-called ‘VMBO’ levels and ‘VMBO/HAVO’ in the Dutch educational system), 22.2% in general higher education (i.e., ‘HAVO’ or ‘HAVO/VWO’), 36.3% in preuniversity education (i.e., ‘VWO’), 3% in secondary vocational education (i.e. ‘MBO’) and 3.4% in higher professional education (i.e. ‘HBO’) or university (i.e. ‘WO’). Mothers were on average 46.17 years of age (*SD* = 4.26; *min* = 35, *max* = 56), while fathers were on average 48.55 years of age (*SD* = 5.64, *min* = 39, *max* = 69). Most mothers and fathers were born in the Netherlands (94.6% and 96.7% respectively) and finished college or university (71.8% and 74.7%, respectively).

### Attrition analysis

To assess potential bias due to missing data, we conducted independent samples t-tests comparing participants who were included in the analytic sample to those excluded due to missingness on one or more variables. Only one significant difference emerged: at baseline, fathers who could not be included in the analyses reported higher frequency of making jokes/sharing funny photos, videos, or texts (*M* = 3.20, *SD* = 0.90) compared to fathers who could be included in our main analyses (*M* = 2.92, *SD* = 0.87; *t*(235) = 2.41, *p* = .017). No other significant differences were detected between these groups.

### Measures

*Depression and anxiety symptoms* of adolescents and parents were measured as an indication of their mental health problems. Depression and anxiety were measured with the ultrabrief Patient Health Questionnaire for Depression and Anxiety [53] that consists of 4 items that cover the 2 core criteria for depressive disorder (e.g., “I had little interest or pleasure in doing things”) and generalized anxiety disorder (e.g., “I felt nervous, anxious or on edge”). The items included a four-point scale (1 = not at all, 2 = several days, 3 = more than half the days, 4 = nearly every day). Composite variables for depressive symptoms and anxiety symptoms were created separately. For each, the mean score of the 2 respective items was calculated, with higher scores indicating higher levels of depression and anxiety.

*Frequency of parent*–*adolescent digital communication* was measured by asking parents ‘how often do you and your child have contact via digital media such as WhatsApp, calling, FaceTime, Snapchat, Skype or Facebook?’ Response options were as follows: 1 = never; 2 = monthly; 3 = weekly; 4 = daily; 5 = several times a day. Parents who responded “never” to this item did not receive the *content of parent*–*adolescent digital contact* items.

*Content of parent*–*adolescent digital communication* included 4 items, each assessing a different content area. Specifically, parents were asked ‘When you and your child have contact via digital media, how often does that contact entail…’: (1) …exchanging practical information, (2) …sharing experiences, (3) …making jokes/sharing funny photos, videos or texts, and (4) …expressing feelings. Parents answered to a five-point Likert scale (1 = never, 2 = seldom, 3 = sometimes, 4 = often, 5 = very often). A higher score indicates more frequent digital communication for that specific content area.

### Analytic plan

A series of hierarchical regression models were conducted in IBM SPSS Statistics (version 29) to assess the main effects of the parent–adolescent digital communication variables (T1) on child’s depression and anxiety symptoms (T2) (step 1), and adolescent’s birth sex as a moderator (step 2). Analyses were conducted using complete cases only (i.e., participants with nonmissing data on all predictors and outcomes). Separate models were conducted for each outcome (depressed mood and anxious mood), and for mothers and fathers (due to the high correlation between mother and father constructs included in the models). In each hierarchical regression model, the outcome (either child’s depressed mood or anxious mood) was first regressed onto the predictors (i.e., all 5 digital communication variables reported by mother/father) and baseline covariates (i.e., adolescent’s depressed/anxious mood, mothers/fathers depressed/anxious mood, adolescent’s age, and gender) (Step 1). In the next step (Step 2), the interaction terms between the 5 digital communication variables (centered) and adolescents’ gender were entered into the model. Given the detected non-normality in the data for depression and anxiety, bootstrapped samples (*N* = 2,000) were employed to estimate the model. The effects were considered significant at *p* < .05 if the asymmetrical 95% confidence intervals (CIs) did not include zero. Simple slopes analysis was conducted to probe significant interaction effects.

## Results

Table 1 presents the descriptive statistics for each measure and the correlations between continuous items, separated by dyad type.

Results from all 4 models are provided in Tables 2 and 3. Only significant effects for our main predictors are reported in-text. However, we do want to highlight here that frequency of overall digital communication was not a significant predictor in any of the models, given the central relevance of this variable to our research questions.

### Mother–adolescent communication predicting adolescent’s depressed mood

Results revealed that the frequency of sharing experiences via digital communication at T1 predicted decreases in adolescent’s depressed mood at T2 (*b* = −0.32, standard error [*SE*] = 0.15, *p* = 0.031, 95% CI [−0.60, −0.02]), and frequency of making jokes and sharing funny photos/videos/texts via digital communication at T1 was associated with increases in adolescent’s depressed mood at T2 (*b* = 0.28, *SE* = 0.12, *p* = 0.017, 95% CI [0.05–0.51]). The interaction between frequency of exchanging practical information via digital communication and adolescent’s gender also significantly predicted adolescent’s depressed mood (*b* = −0.50, *SE* = 0.23, *p* = 0.030, 95% CI [−0.97, −0.07]) (see Table 2).

**Figure 1.**
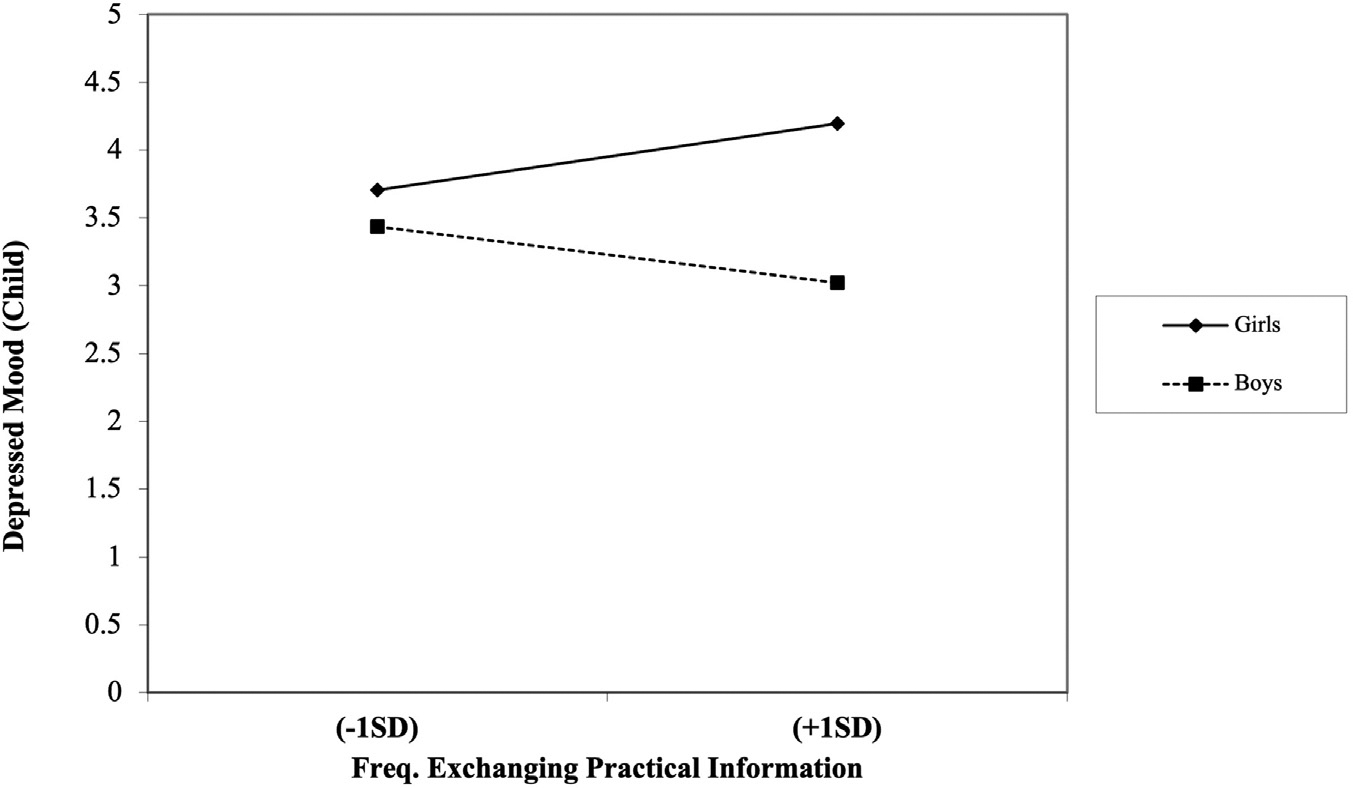
Interaction between frequency of exchanging practical information and adolescent’s sex on child’s depressed mood.

Simple slopes analysis revealed a significant positive association between frequency of exchanging practical information (T1) and depressed mood (T2) for girls (*b* = 0.27, *t* = 2.28, *p* = 0.024) (see Figure 1). In contrast, the relationship between the frequency of exchanging practical information and depressed mood for boys was negative; however, that association was only trending toward significance (*b* = −0.23, *t* = −1.85, *p* = 0.066).

### Mother–adolescent communication predicting adolescent’s anxious mood

Results revealed that the frequency of sharing experiences via digital communication (T1) predicted decreases in adolescent’s anxious mood (T2) (*b* = −0.43, *SE* = 0.16, *p* = 0.006, 95% CI [−0.73, −0.12]) (see Table 2).

### Father–adolescent communication predicting adolescent’s depressed mood

Results revealed that the frequency of making jokes and sharing funny photos/videos/texts via digital communication (T1) was associated with increases in adolescent’s depressed mood (T2) (*b* = 0.38, *SE* = 0.15, *p* = .014, 95% CI [0.08–0.70]) (see Table 3).

### Father–adolescent communication predicting adolescent’s anxious mood

Results revealed that the frequency of exchanging practical information via digital communication (T1) predicted increases in adolescent’s anxious mood (T2) (*b* = 0.29, *SE* = 0.13, *p* = 0.021, 95% CI [0.03–0.54]) (see Table 3).

## Discussion

Findings from this study reveal that the relationship between parent–adolescent digital communication and subsequent adolescent-reported depression and anxiety symptoms was not related to the frequency of overall digital communication. Instead, it was related to the frequency of specific communication content. Specifically, exchanging experiences with mothers seems protective against depression symptoms for both sons and daughters, and sharing practical information with mothers seems protective against depression symptoms for sons, but not for daughters. Additionally, our findings suggest that greater frequency of making jokes/sharing funny photos, videos, or texts was associated with greater depression symptoms at follow-up, while exchanging practical information with fathers was associated with higher levels of anxiety symptoms. Notably, all these effects were independent of the parents’ depression and anxiety symptoms and the age of the child. Taken together, these findings highlight the nuanced relationship between the content of digital communication between parents and adolescents and the mental health outcomes of the latter. The type of the content shared appears to have more significant implications for adolescent mental health than the sheer volume of communication, underscoring the importance of focusing on the quality and context of parent–adolescent interactions in the digital realm as potential areas for intervention and support in mental health strategies.

Previous research offers some explanations for the current findings. First, the protective effects of sharing experiences on adolescent depression symptoms may be in part due to bonding that can occur when the family shares events and memories [54]. These bonding experiences may lead to increased trust and information sharing by the adolescent that could contribute to better parent–adolescent relationships and increased monitoring, both of which are known protective factors against depressive symptoms [20,34,55]. Together, these factors highlight the importance of shared experiences in fostering emotional connections that may help protect adolescents from depressive symptoms.

Next, prior research also offers some explanations for why more frequent exchanges of practical information had negative effects on daughters’ depression symptoms when these exchanges were with mothers, as well as why this type of communication exchange with fathers had a negative effect on both daughters’ and sons’ anxiety symptoms. One plausible explanation is that frequent practical communication may be perceived as overly controlling. While occasional exchanges of practical information, such as when to come home for dinner, may not be harmful, frequent exchanges could contribute to a sense of excessive parental monitoring [31,34], a characteristic commonly associated with authoritarian parenting. Authoritarian parenting, which is more often associated with fathers than mothers [50], has been linked to internalizing symptoms, such as depression and anxiety [56]. Related to perceived control, the negative effects of practical information exchanges with mothers on daughters’ depression may stem from daughters being more reactive to their mothers than sons are [57]. This heightened sensitivity may result in daughters perceiving these interactions differently, which could explain the observed effects. Indeed, given the preliminary nature of these findings, future studies are needed to further explore the reasons behind these gender differences.

Finally, the negative effects of parent–adolescent exchanges of jokes or funny photos, videos, or texts on depressive symptoms may be attributable to humor as a coping mechanism for depression. Previous research suggests that humor is indeed a way that adolescents and adults cope with depressed states [58,59], with studies also linking positive effects of humor related coping between mother–daughter dyads [22,45]. Notably, Pantaleao & Ohannessian (2018) found that while boys utilized humor-related coping as a strategy more than girls did, they derived no benefit in terms of their depression/anxiety symptoms. This suggests that the type of humor may be an important caveat to this finding, as girls may utilize humor that is aimed at reducing interpersonal tension, whereas boys often use humor that is more aggressive and darker in nature [60]. It is additionally important to note that humor, the content and context of which is so deeply entrenched in culture, may be tricky to generalize across international populations. Based on the current findings, however, one might conclude that humorous exchanges between parents and their children, which may provide the illusion of improvements in adolescents’ depressive states to parents, are counterproductive in the long-term. Utilizing humor with an adolescent who is experiencing depressive/anxiety symptoms may perceive these attempts as invalidating their experiences or attempting to ignore the issue. Alternatively, adolescents who frequently utilize humor may be doing so to mask negative feelings. Due to the bidirectional nature of the communication assessed it is not known from the current study if parent- or adolescent-initiated humor is more detrimental than the other. Ultimately, future studies are needed to further understand the nature of these effects, though parents who engage frequently in these types of exchanges may still benefit from knowing that the use of humor may be covering deep-rooted issues that can only be addressed through professional help.

### Implications

Findings from this study shed some light on how the nature of content shared through digital communication between parents and adolescents may influence depression and anxiety symptoms. This highlights the importance of the frequency of exchanging certain content over the overall frequency of digital interactions. The differential effects based on the content of communication—whether sharing practical information or humorous messages—underscore the importance of considering the quality and context of digital contact in future studies. While direct application to practitioner advice may be premature, our findings can further inform theoretical frameworks by delineating the types of digital interactions that are associated with better or worse mental health outcomes. Specifically, the nuanced effects of digital communication content suggest that interventions aiming to leverage or mitigate digital interactions’ impact on adolescent well-being need to be finely tuned to the nature of the exchanged content. This underscores an emerging need for models of adolescent mental health that integrate many aspects of digital communication with parental figures, specifically attending to the varied effects of different communication content.

### Limitations and Future Directions

Despite the valuable insights from this study, there are several limitations that warrant attention in future research. First, the exchanges assessed here do not allow us to probe whether the frequency of these interactions was primarily initiated by the parent or the adolescent. Additionally, the exact content and tone of specific digital exchanges were not examined, and these factors may be important; for instance, parents who use a warmer tone when digitally communicating with their adolescent may elicit a different response than parents who do not [29]. Future studies are therefore needed to distinguish between the roles of initiation and tone in these exchanges.

Another limitation is that we did not assess who, when, and why parents and their adolescents initiated digital exchanges. Given the bidirectional nature of the communication questions, where parents reported instances when either they or their adolescent-initiated conversations, it is possible that not all instances were fully remembered or accurately captured. Relying on parent reports may lead to underestimations of the true frequency and nature of these interactions due to potential biases. However, the significant associations we observed with parent reports imply that even with such underestimations, parental perceptions are meaningful predictors of adolescent internalizing symptoms. Future studies should employ more precise measures or mixed-method approaches to better document these nuances (e.g., logging data directly from smartphones, using both parent and adolescent reports).

An additional limitation is that we were unable to directly compare mother and father effects, due to data constraints. Future research with larger or more balanced samples is needed to explore potential differences between maternal and paternal influences on adolescent mental health. Also, although we used a 12-month interval between waves, we know that the development of anxiety/depression may take years [37]. This highlights the need for a larger longitudinal study with additional waves to better investigate the potential mechanisms underlying the relationship between parent–adolescent digital communication and adolescent well-being.

Finally, the absence of in-person interactions is another limitation of this study that should be addressed in future research, given that face-to-face communication may interact with digital exchanges to influence adolescent mood states in unique ways. We also did not include other potential individual and social risk and protective factors that may contribute to or prevent adolescent internalizing symptoms and mood states (e.g., socialization influences of parent depression/anxiety through modeling or impaired parenting, digital communications with peers). This is particularly relevant since an accumulation of risk factors may contribute to the development of depression and anxiety symptoms in adolescents [61]. Including a more comprehensive set of these factors in future studies would provide a deeper understanding of how parent–adolescent digital exchanges fit within a broader developmental context and impact adolescent mental health.

## Figures and Tables

**Table 1:** Descriptives and correlations for continuous variables

	Variables	1	2	3	4	5	6	7	8	9	10	11	12
1	Adolescent age at baseline	–	0.150	**0.170** *	0.004	−0.033	−0.086	0.090	0.007	0.040	0.144	0.113	0.063
2	Contact through digital media such as WhatsApp, calling, FaceTime, Snapchat, Skype, or Facebook	**0.257** **	–	0.147	**0.438** **	**0.376** **	**0.271** **	**0.340** **	0.020	**0.188** *	**0.192** *	0.152	**0.274** **
3	Exchanging practical information (e.g., asking or telling each other what time you’ll be home for dinner)	**0.214** **	**0.252** **	–	0.126	0.037	0.060	0.026	0.062	0.164	−0.117	0.123	**0.197** *
4	Sharing experiences (e.g., sending a photo or message about a situation you or your child is experiencing)	−0.010	**0.357** **	**0.231** **	–	**0.657** **	**0.386** **	0.140	0.091	**0.233** **	0.101	**0.231** **	**0.233** **
5	Making jokes/sharing funny photos, videos, or texts	0.004	**0.259** **	**0.188** **	**0.610** **	–	**0.388** **	0.020	0.144	**0.293** **	0.137	**0.257** **	**0.316** **
6	Expressing feelings (e.g., talking or asking about how you’re doing)	0.141	**0.448** **	**0.324** **	**0.545** **	**0.447** **	–	−0.046	0.000	0.090	0.005	0.026	0.116
7	Parent baseline depressive symptoms	0.041	−0.114	−0.022	−0.086	0.042	0.046	–	0.024	0.165	**0.646** **	0.144	**0.247** **
8	Adolescent baseline depressive symptoms	0.105	−0.029	−0.028	0.006	0.129	**0.153** *	0.005	–	**0.526** **	0.075	**0.616** **	**0.457** **
9	Adolescent follow-up depressive symptoms	0.106	0.070	0.034	0.004	**0.174** *	**0.161** *	−0.062	**0.542** **	–	**0.206** *	**0.467** **	**0.740** **
10	Parent baseline anxiety symptoms	0.003	0.009	−0.096	−0.124	0.065	0.034	**0.574** **	−0.045	−0.085	–	**0.179** *	**0.240** **
11	Adolescent baseline anxiety symptoms	0.155	0.010	0.031	0.057	0.126	0.103	−0.033	**0.602** **	**0.489** **	−0.019	–	**0.596** **
12	Adolescent follow-up anxiety symptoms	0.081	0.004	0.083	−0.042	0.120	0.103	−0.035	**0.466** **	**0.764** **	−0.009	**0.582** **	–
Mother–Adolescent	M (SD)	13.87 (2.02)	3.59 (0.86)	4.03 (0.91)	3.27 (0.96)	3.07 (1.09)	2.72 (1.13)	2.73 (0.99)	3.36 (1.43)	3.66 (1.68)	3.27 (1.25)	3.45 (1.68)	3.73 (1.79)
Father–Adolescent	M (SD)	14.04 (2.00)	3.46 (0.89)	3.96 (0.83)	3.01 (0.85)	2.93 (0.86)	2.43 (0.97)	2.75 (1.21)	3.37 (1.45)	3.59 (1.70)	2.95 (1.47)	3.42 (1.62)	3.64 (1.70)

Mother correlations are under the diagonal, father correlations are above the diagonal. Significant correlations are in bold.

* *p* < .05.

** *p* < .01.

**Table 2:** Results from regression models assessing the effects of digital communication between mother and adolescent on depression (left panel) and anxiety (right panel) symptoms

Predictors (T1)	T2 – Depression symptoms (adolescent)					T2 – Anxiety symptoms (adolescent)				
	b	SE	*p*	95% CI		b	SE	*p*	95% CI	
				*LB*	*UB*				*LB*	*UB*
Step 1										
Mother-Child Digital Communication										
Frequency of Exchanges	0.08	0.14	.552	−0.21	0.34	−0.02	0.12	.856	−0.26	0.20
Freq. Exchanging Practical Information	0.01	0.12	.948	−0.22	0.24	0.13	0.13	.321	−0.12	0.39
Freq. Sharing Experiences	**−0.32**	**0.15**	**.031**	**−0.60**	**−0.02**	**−0.43**	**0.16**	**.006**	**−0.73**	**−0.12**
Freq. Making Jokes & Sharing Funny Media/Texts	**0.28**	**0.12**	**.017**	**0.05**	**0.51**	0.24	0.13	.068	−0.03	0.50
Freq. Expressing Feelings	0.07	0.13	.561	−0.18	0.34	0.10	0.12	.427	−0.14	0.34
Covariates										
Depressive | Anxious Symptoms (Adolescent)	**0.54**	**0.08**	**<.001**	**0.37**	**0.69**	**0.51**	**0.09**	**<.001**	**0.34**	**0.68**
Depressive | Anxious Symptoms (Mother)	−0.16	0.10	.078	−0.34	0.04	−0.05	0.09	.610	−0.24	0.14
Age (Adolescent)	0.04	0.06	.565	−0.07	0.16	−0.01	0.06	.811	−0.13	0.11
Gender (Adolescent)	**−0.72**	**0.21**	**<.001**	**−1.14**	**−0.31**	**−0.77**	**0.23**	**.004**	**−1.20**	**−0.31**
Step 2										
Interactions										
FE * Gender	0.30	0.25	.236	−0.17	0.84	−0.15	0.25	.554	−0.64	0.38
FEPI * Gender	**−0.50**	**0.23**	**.030**	**−0.97**	**−0.07**	−0.04	0.28	.889	−0.63	0.48
FSE * Gender	−0.10	0.29	.728	−0.69	0.44	0.34	0.33	.290	−0.33	0.97
FMJ * Gender	0.01	0.25	.967	−0.46	0.52	−0.23	0.28	.403	−0.76	0.35
FEF * Gender	−0.12	0.25	.655	−0.64	0.36	0.01	0.25	.980	−0.50	0.48

Significant correlations of *p* < .05 are in bold.

CI = confidence interval; FE = frequency of exchanges; FEF = frequency of expressing feelings; FEPI = frequency of exchanging practical information; FMJ = frequency of making jokes; FSE = frequency of sharing experiences; LB = lower boundary; UB = upper boundary.

**Table 3:** Results from regression models assessing the effects of digital communication between father and adolescent on depression (left panel) and anxiety (right panel) symptoms

Predictors (T1)	T2 – Depression symptoms (adolescent)					T2 – Anxiety symptoms (adolescent)				
	b	SE	*p*	95% CI		b	SE	*p*	95% CI	
				LB	UB				LB	UB
Step 1										
Father-Child Digital Communication										
Frequency of Exchanges	0.03	0.14	.793	−0.27	0.28	0.20	0.13	.115	−0.04	0.45
Freq. Exchanging Practical Information	0.22	0.13	.086	−0.02	0.50	**0.29**	**0.13**	**.021**	**0.03**	**0.54**
Freq. Sharing Experiences	0.00	0.15	.981	−0.30	0.30	−0.18	0.16	.256	−0.50	0.14
Freq. Making Jokes & Sharing Funny Media/Texts	**0.38**	**0.15**	**.014**	**0.08**	**0.70**	**0.32**	**0.17**	**.050**	**−0.004**	**0.66**
Freq. Expressing Feelings	0.00	0.13	.985	−0.24	0.27	0.05	0.12	.687	−0.17	0.30
Covariates										
Depressive | Anxious Symptoms (Adolescent)	**0.53**	**0.09**	**<.001**	**0.33**	**0.70**	**0.47**	**0.11**	**<.001**	**0.26**	**0.68**
Depressive | Anxious Symptoms (Father)	0.17	0.13	.198	−0.11	0.42	0.17	0.09	.059	−0.01	0.35
Age (Adolescent)	0.02	0.06	.809	−0.11	0.14	−0.03	0.06	.637	−0.16	0.09
Gender (Adolescent)	**−0.54**	**0.26**	**.038**	**−1.06**	**−0.03**	−0.54	0.27	.044	−1.04	−0.02
Step 2										
Interactions										
FE * Gender	−0.27	0.29	.350	−0.82	0.35	−0.27	0.26	.305	−0.74	0.29
FEPI * Gender	−0.01	0.28	.987	−0.54	0.56	−0.08	0.26	.757	−0.54	0.45
FSE * Gender	0.09	0.37	.802	−0.66	0.81	0.22	0.36	.521	−0.50	0.93
FMJ * Gender	0.12	0.36	.731	−0.58	0.86	−0.27	0.37	.473	−1.00	0.46
FEF * Gender	−0.11	0.28	.681	−0.69	0.43	0.19	0.24	.428	−0.27	0.66

Significant correlations of *p* < .05 are in bold.

CI = confidence interval; FE = frequency of exchanges; FEF = frequency of expressing feelings; FEPI = frequency of exchanging practical information; FMJ = frequency of making jokes; FSE = frequency of sharing experiences; LB = lower boundary; UB = upper boundary.

## Data Availability

The dataset associated with this study is not currently available online.
